# Brain AVMs: An Endovascular, Surgical, and Radiosurgical Update

**DOI:** 10.1155/2014/834931

**Published:** 2014-10-21

**Authors:** Simone Peschillo, Alessandro Caporlingua, Claudio Colonnese, Giulio Guidetti

**Affiliations:** ^1^Department of Neurology and Psychiatry, Endovascular Neurosurgery/Interventional Neuroradiology, “Sapienza” University of Rome, Viale del Policlinico 155, 00100 Rome, Italy; ^2^Department of Neurology and Psychiatry, Neurosurgery, “Sapienza” University of Rome, Rome, Italy; ^3^Department of Neurology and Psychiatry, Neuroradiology, “Sapienza” University of Rome, Italy; ^4^Department of Neurology and Psychiatry, Interventional Neuroradiology, “Sapienza” University of Rome, Italy

## Abstract

Brain arteriovenous malformations (bAVMs) are complex vascular lesions. Despite multiple studies, several classifications, and a great interest of the scientific community, case selection in AVM patients remains challenging. During the last few years, tremendous advancements widened therapeutic options and improved outcomes spreading indications for patients harboring lesions deemed inoperable in the past. Anatomical and biological case specific features, and natural history with a focus on presenting symptoms should be evaluated case by case and always kept in mind while planning a therapeutic management for a bAVMs. A multidisciplinary approach is strongly recommended when dealing with bAVMs and should involve physicians expertise in this kind of challenging lesions. The goal of this paper is to provide a focused review of the most recent acquisitions and therapeutic strategies regarding surgical, endovascular, and radiosurgical treatment.

## 1. Introduction

Brain arteriovenous malformations (bAVMs) structure consists of a primary pial arterial supply converging into a nidus directly connected to a venous drainage network without interposition of a capillary bed. Blood flow in bAVM is turbulent, characterized by high velocities and low resistances completely ignoring the natural equilibrium dictated by cerebral hemodynamic autoregulation. Other peculiar features include a tendency to bleed and to develop vascular anomalies such as aneurysms and venous ectasia [1].

Clear data concerning bAVM epidemiology are still unavailable although an estimation of their incidence is set on values between 0,001% and 0,5% [1, 2]. At least 1-2% of all the hemorrhagic strokes are the result of a bleeding bAVM. Among patients harboring such vascular lesion, 12% will experience symptoms during their lifetime [2]. The annual hemorrhagic risk for unruptured bAVM reaches 2%–4% [3]; following a first rupture event this risk increases, achieving 6%–8% during the first year and then it drops to the aforementioned initial values [1]. Morbidity and mortality are fixed around, respectively, 80% and 10%–30% [4, 5].

Etiopathogenesis of bAVM is controversial: some authors theorized how they may develop during intrauterine life and some others advocated an angiopathic reaction following either a cerebral ischemic or hemorrhagic event as the “primum movens” of their development [1]. In 2009, Kim et al. [6] advocated the activity of matrix metalloproteinases (MMP-9) and antigenic and inflammatory factors, such as interleukin-6, as a possible cause of bAVM rupture. More specifically, being a proteolytic enzyme, MMP-9 demolishes proteins found in the extracellular matrix (ECM) and it has been found at high concentrations in bAVM nidus. Therefore MMP-9 is probably involved in a series of alterations ultimately leading to vessels wall fragility and thus higher susceptibility to rupture [7]. bAVMs are extremely frequent in certain group of patients afflicted by particular hereditary diseases such as the hereditary hemorrhagic telangiectasia, Parkes-Weber syndrome, Wyburn-Mason syndrome, and Cobb syndrome. The location of a bAVM influences profoundly its hemorrhagic risk; indeed deep seated lesions in the proximity of the basal ganglia or in the periventricular regions are more likely to bleed. Moreover the presence of a deep venous drainage, associated aneurysms or venous varices, and venous stenosis or arteriovenous fistula are other factors contributing to an even higher risk of hemorrhage [4, 8]. Conversely, accordingly to a recently published meta-analysis [8], male sex, small brain AVMs, and those with strictly deep venous drainage were associated with lower case fatality.

## 2. Treatment Options

Numerous classifications have been proposed in an effort to provide neurosurgeons and neurointerventionists with a reliable instrument to support therapeutic choices when dealing with bAVM. In 1986, Spetzler and Martin [7] introduced a classification systems based on dimensions, location, and type of venous drainage which estimated surgical risk for bAVMs (Table 1). Subsequently in 2011, Spetzler himself and Ponce proposed a 3-category classification for bAVM [10]. Lawton et al. [11] brought their contribution including variables such as patient age, hemorrhagic presentation, nidal diffuseness, and deep perforating arterial supply. Despite those efforts, an adequate classification is still unavailable mostly due to the fact that the complexity of bAVM hampers any attempt of simplification using parameters either arbitrary chosen or selected on the basis of their statistical significance. Factors such as the presence of intranidal or perinidal aneurysms, varicose or stenotic veins, turbulent flow, pial fistula, or wall-shear stress may not be as decisive as the currently used parameters for bAVM classification; nonetheless they ultimately influence bAVM hemorrhagic risk. These classifications have been used in the past and partly are still being used nowadays to support bAVM therapeutic management.

bAVM therapeutic approach is multidisciplinary: a cure may be achieved thanks to surgery, radiosurgery, neurointerventional surgery, or a combination of the three. More specifically, the endovascular management for bAVM may include interventions providing a reduction of bAVM nidus dimension in a presurgical setting, reduction prior to radiosurgery, or elimination of certain, often associated, vascular anomalies such as aneurysms, venous varices, and fistulas. Supporting evidences on the behavior of bAVMs managed conservatively performed through mere observation and radiological follow-up may be a viable option considering the risk of complications entailed with the treatment of these complex and unpredictable lesions. A recently published clinical trial called ARUBA [12] (a randomised trial of unruptured brain arteriovenous malformations) brought decisive evidences toward a more “cautious” attitude concerning bAVMs treatment; however, the study presented some weaknesses, among which the lack of distinction between different kinds of treatment (patients randomized in the “interventional” group were mostly treated via the endovascular route, which is known to be keen to complications) and the investigators themselves advocated the need of further research and follow-up in order to confirm their findings. Details on ARUBA are reported at the end of the present review paper for the purpose of completeness (see the appendix).

## 3. Microsurgical Treatment

The gold-standard in bAVM management is surgery whenever viable. According to published patient series, in 94%–100% of the cases an angiographic cure with low morbidity rates (from 1% to 10%) can be obtained in small (nidus < 3 cm) bAVMs when surgery is performed by expert vascular neurosurgeons [1]. These percentages greatly vary when dealing with bigger lesions located in critical or eloquent brain regions: for instance in IV and V grade bAVMs, according to Spetzler and Martin classification, an angiographic cure may be obtained only in, respectively, 22% and 17% of the cases [1]. According to Hartmann et al. [13], during a long conducted follow-up in surgically treated patients, 3% sustained disabling neurologic deficits versus 32% of treatment-related new nondisabling neurological deficits. A recently published meta-analysis described complications leading to permanent neurological deficits or death occurring in a mean of 7.4% (range, 0%–40%) patients after microsurgery; successful brain AVM obliteration was achieved in 96% (range, 0%–100%) patients with this kind of treatment [8]. Surgery may be part of a multimodal therapeutic management involving a preliminary endovascular approach in order to reduce nidus volume and curing or mitigating eventual additional vascular anomalies as the ones already mentioned. Most often, neurosurgeons expect from their neurointerventional colleagues a selective embolization of the deep arterious feeders sited in the opposite site of the surgical operation field prioritizing the use of Onyx rather than glue to improve surgical resectability.

Interesting results have been drawn from studies focusing on patients treated with a combination of surgery and radiosurgery [11]: the rationale behind this type of approach lies on the possibility to surgically approach bAVMs, previously deemed inoperable, after either a partial or global reduction of their dimensions through radiosurgery. Conversely the latter may be used as an adjuvant therapy after surgery.

Tremendous improvements of the neurosurgeon armamentarium have further widened the range of therapeutic options. Killory et al. [15] described how the use of green indocyanine would allow visualizing residual portions of bAVMs during surgery maximizing surgical outcomes. Moreover a drastic technical amelioration has been brought by the introduction of nonstick bipolar forceps and Thulium laser. The effects of adenosine, such as asystole, known to be used during surgery for aneurysms, have found new applications for the management of bAVMs, especially when trying to reach deep sited portions of the lesion or to deal with massive intraoperative bleeding. The recent breakthrough of newly acquired 3D technology applications in surgery may soon allow real-time fusing of angiographic or MRI images with the surgical operation field playing a synergic role with other imaging tools such as neuronavigation, tractography, and functional MRI to further boost progress toward a better understanding of bAVMs anatomy and consequently better surgical outcomes.

## 4. Endovascular Treatment

Even though the final goal of bAVM therapy is the complete obliteration of its nidus, this is not always possible. An endovascular approach may be adopted in at least five possible scenarios: indeed an embolization may be performed either prior to surgery/radiosurgery, or to treat vascular anomalies with a bAVM, as a curative therapy or in a palliation setting (i.e., mitigation of blood flow steal symptoms) [14]. Embolic agents are numerous, each with peculiar advantages and limitations. Among the most commonly used n-butyl cyanoacrylate (n-BCA), ETOH, PVA/Embospheres, coils and, more recently, Onyx, a biocompatible polymer of ethyl vinyl alcohol copolymer dissolved in an organic solvent (dimethyl sulfoxide) which allows significant AVM volume reduction and, in some cases, angiographic and anatomic cure [16] (Table 2). The introduction of microcatheters equipped with detachable tips (Apollo, Covidien, USA; Sonic, Balt, France) changed radically the techniques used to inject embolic materials as these devices are far less susceptible to complications due to entrapment of the microcatheter itself allowing better efficacy of the endovascular treatment. Unfortunately, at present, these microcatheters with detachable tips are still unavailable in the United States.

Despite the intra-arterial route is the most commonly used, during the last few years a transvenous approach has been developed with reported good results so far [17, 18]. Traditionally, endovascular neurosurgeons have always been reluctant when considering such an option mostly due to the risk of hemorrhagic complications derived from any manipulation of the venous side of a bAVM when its core is still patent. The transvenous route can be chosen only in selected cases, for instance when it is not possible to navigate the microcatheter through small and tortuous arteries to reach the AVM nidus, in case of high-flow venous side aneurysm occlusion or when surgery or radiosurgery is not viable. Two aspects of the endovascular transvenous approach are worth citing here: the progressive and controlled lamination of Onyx within the draining vein and the transvenous rapid nidal occlusion with the retrograde filling of all its arterial feeders, which may prevent hemorrhagic complications. In a swine experimental study, Massoud [19] showed how induced hypotension would support a transvenous embolization: indeed it allows the embolic material to reach retrogradely the arterial side with more ease providing better results in terms of nidal obliteration for AVM.

An innovative injection technique for Onyx has been recently proposed by Chapot et al. [20] and presented under the name of “pressure-cooker” technique. Normally, good results with Onyx injection are obtained by creating a plug in the vascular lumen just proximal to the microcatheter tip and then start injecting. In Chapot's technique an antireflux plug is created by trapping the detachable part of an Onyx-compatible microcatheter with coils and glue in order to obtain wedge-flow conditions, thereby enabling a better understanding of macrofistulous AVMs and a more comprehensive, forceful, and controlled Onyx embolization. With the same rationale, double lumen balloons (Scepter XC, Microvention, USA) may play a role in bAVMs endovascular therapy; more specifically the inflation of a balloon proximal to the Onyx injection site may avoid the need for a plug and its associated risks. Main limitation of this technique lies in the difficulty encountered while navigating the dual-lumen balloon into distal arterial feeders, especially small ones because these vessels can often be accessed only with flow-directed microcatheters or small microcatheters and an overpenetration of the nidus with negative hemodynamic consequences, especially with venous penetration [21].

Regarding morbidity and mortality of an endovascular approach, complications leading to permanent neurological deficits or death occurred in 6.6% (range, 0%–28%) after embolization while successful bAVM obliteration occurred in 13% cases (range, 0%–94%) [8]. Future advancements in bAVMs endovascular management may be brought by new embolic materials with peculiar features specifically conceived, for example, to reduce radiopacity after their use and thus allowing better visualization during following endovascular procedures if needed.

## 5. Radiosurgical Treatment (RS)

Radiosurgery has been building its success in selected cases of high grade bAVMs (mostly IV and V grade lesions according to Spetzler and Martin classification) considered inoperable or highly prone to severe or even fatal complications if treated with other kinds of approaches. Radiosurgery retains at least two limitations: the latency of postoperative results and the iatrogenic morbidity. Latency for devascularization results reaches usually 2 years' time from radiosurgery (can be up to 4 years), a period during which patients are unfortunately exposed to hemorrhagic risks comparable to nonoperated patients with comparable vascular lesions. The second limitation concerns the structures adjacent to the radiosurgical target volume which may be affected by radiations, leading to an iatrogenic morbidity.

Complete obliteration is observed in 50%–90% of the cases and its rate is inversely proportional to the bAVM nidus size [5, 22, 23]. Once considered as cured, a hemorrhagic event may occur in less than 1% of patients [24]. Radiation dosage and correct interpretation of the malformation anatomy have a decisive influence on cure percentages: one should constantly keep into account both risks and benefits when choosing dosage/volume ratios, tailoring each procedure case by case and striving to reach, at least, a <3% risk of perilesional tissular damage [25]. A thorough evaluation of benefits and risks must be conducted before choosing the selected variables. MRI and angiography are the gold standard for treatment planning: to achieve the best result, occlusion of the malformation with minimal risk of adverse events, it is of paramount importance to precisely locate the arteriovenous shunt. Moreover isodose areas corresponding to perilesional tissues should be 50% to 80% less than those centered to the AVM core in order to minimize iatrogenic morbidity [26].

Radiosurgery may be burdened by complications leading to permanent neurological deficits or death in 5.1% (range, 0%–21%) of the cases [8]. In an effort to reduce the likelihood of such adverse events, a pharmacological therapy including steroids, pentoxifylline, and vitamin E may be adopted; in selected cases anticoagulant drugs, barbiturates, hypothermia, or hyperbaric oxygen therapy may be used [25]. 21-aminosteroids have shown their beneficial effects providing inhibition of lipid peroxidation triggered by induced free radicals, known to cause cellular and, ultimately, vascular damage; unfortunately though, they do not influence cellular reactions involved in the necrotic process.

Even though bAVMs embolization often precedes radiosurgery, recently acquired data have shown how such a conduct may worsen radiosurgical outcomes: indeed obliteration percentages in patients receiving partial embolization prior to radiosurgery are definitely worse than patients harboring nonoperated malformations [25]. The pathophysiological mechanism behind this evidence is still obscure; probably the occlusion of the main arterial feeders with the smaller ones left untouched may lead to an angiogenetic reaction triggered by ischemia. Moreover a partial embolization may induce recruitment of new vascular subsystems thereby crippling radiosurgery benefits [25]. Furthermore, Onyx (Covidien, Irvine CA), whose popularity is increasingly growing as the embolic material of choice for vascular malformations management, disrupts radiosurgery planning stages as its strong radiopacity creates radiological artifacts preventing acquirement of a complete understanding of bAVMs anatomy which is, as already stated above, of the utmost importance for the success of the procedure. bAVMs presenting with pial fistulas retain a superior hemorrhagic risk and appear to be refractory to radiosurgery; those associated with extra or intranidal aneurysms are more likely to bleed with a 5-years 10-fold estimated hemorrhagic risk if compared with bAVMs not presenting with such additional vascular anomalies [25]. For such complex cases, as for bAVMs in general, a multidisciplinary approach is strongly recommended.

*Appendix: ARUBA*. The recently published ARUBA study [12] is a randomized study that evaluates the risks of treatment of unruptured arteriovenous malformations compared to those implied by a conservative management. Adult patients (≥18 years) with an unruptured brain arteriovenous malformation were enrolled into this trial at 39 clinical sites in nine countries. Patients were randomised to medical management with interventional therapy (i.e., neurosurgery, embolisation, or stereotactic radiotherapy, alone or in combination) or medical management alone (i.e., pharmacological therapy for neurological symptoms as needed).

The study, started on April 4, 2007, was stopped in advance on April 15, 2013, because the increased risk of treatment was evident compared to natural history. This study is based on the assumption that the natural history of intact AVMs is less dangerous than commonly considered; therefore the risks of any treatment are not justified. This study has raised several criticisms: the main one concerns the nature of the treatment that has been compared with natural history: there is no distinction between the kind of treatment, which was mostly endovascular, a treatment that is already known to imply several complications. On a case study of 223 patients evaluated, of its planned 400, 114 assigned to interventional therapy and 109 to medical management, only 18 were treated surgically. Mean follow-up was 33 months. The primary outcome of death or stroke was seen in 11 patients (10%) in the conservative group and 39 patients (29%) in the interventional group. However, the long-term results remain unknown. The ARUBA investigators plan to continue to follow up this group to determine whether the differences persist.

The study also confirms that the risk of bleeding in case studies collection is around 3% per year in unruptured malformations.

## 6. Conclusions

Innovation defined by the technological development including new embolic materials, catheters, and techniques resulted in a safer and more effective treatment of brain AVMs. In order to obtain the best therapeutic outcomes, a multimodal, case-tailored approach should be adopted. Anatomical and biological case specific features, natural history with a focus on presenting symptoms should be evaluated case by case and always kept in mind while planning a therapeutic management for bAVMs. All patients should be evaluated by physicians with expertise in endovascular embolization, microneurosurgical resection, and radiosurgery. Despite multiple studies, several classifications and a great interest by the scientific community, case selection in AVM patients remain challenging.

## Figures and Tables

**Table 1:** Grading systems for AVMs.

Spetzler-Martin grading system for AVMs [9]		Spetzler-Ponce grading system for AVMs [10]			Grading score proposed by Lawton [11]	
	Points	Class	Spetzler-Martin grade			Points
Size of nidus					Age (years)	
Small (<3 cm)	1	A	I, II	Surgical resection	<20	1
Medium (3–6 cm)	2	B	III	Multimodality treatment	20–40	2
Large (>6 cm)	3	C	IV, V	No treatment	>40	3
Location					Unruptured presentation	
Noneloquent site	0				No	0
Eloquent site	1				Yes	1
Pattern of venous drainage					Diffuse	
Superficial only	0				No	0
Deep	1				Yes	1

**Table 2:** Embolic materials.

Embolic material	Advantages	Limitations
N-butyl cyanoacrylate (n-BCA)	(i) Great penetration potential into bAVMs nidus. (ii) Permanent embolization with durable occlusion of the embolized vessel or pedicle. (iii) Deliverable through small, flexible, and flow-directed catheters causing minimal trauma even in distal vessels of the cerebrovascular system. (iv) Easy and quick delivery, infusion generally takes less than 1 minute. (v) Radiolucent, must be mixed with a radiopaque agent (i.e. ethiodized oil: lipiodol, ethiodol). Usual ratios for the mixture are 1.5 : 1 to 3 : 1 (oil-to-NBCA) with nonnegligible margin of error. (vi) Radiolucent. Follow-up angiograms and eventual indications for further endovascular surgery are not hampered by radiological artifacts from the first intervention.	(i) Experience is required to judge the best fitted ratio for NBCA/Ethiodol for each different scenario. (ii) Adhesive-tendency to adhere to the catheter, making withdrawal traumatic or impossible. (iii) High level of expertise is required to control the injection to achieve adequate nidal obliteration preventing venous dissemination. (iv) Far higher consistency than ONYX. In case of packed AVM it cannot be removed piecemeal with scissors.

Onyx	(i) Nonadhesive, (ii) Great radiopacity—enhanced angiographic control during injection. (iii) Lesser consistency than NBCA. In a packed AVM can be removed piecemeal with scissors.	(i) DMSO component of the mixture may induce vasospasm and angionecrosis. (ii) Tantalum powder must be mixed with the agent to provide radiopacity. (iii) Great radiopacity—follow-up angiograms and eventual subsequent endovascular procedures are hampered by radiological artifacts.

Ethanol (ETOH)	(i) Sclerosant-dehydration and disruption of endothelium surface with fractures of the vessel walls to the level of the internal elastic lamina resulting in acute thrombosis. (ii) Great penetration potential.	(i) Risk of significant brain edema. (ii) It may induce pulmonary precapillary vasospasm possibly leading to cardiopulmonary collapse. (iii) Great penetration potential-high level of experience is required to perform ETOH embolization safely.

Polyvinyl alcohol (PVA)/Embospheres	(i) Penetration potential depends on particle size allowing the adoption of different strategies in function of case specific angiographical features. (ii) Once injected particles expand obstructing vessels with higher diameters than the catheters. (iii) Particles are far more controllable than embolic liquid agents during injection.	(i) Particulate embolization requires a microcatheter with an internal diameter larger than the particle itself. (ii) During mixing process, PVA particles may fragment contaminating the mixture with smaller “dangerous” emboli. (iii) Risk of particles to clump up and/or catheters to be clogged due to particles high friction coefficient. Potential risk of vascular perforation. (iv) The choice of the particles' size depends on operator's interpretation of the superselective angiogram. (v) Nonpermanent embolization effects—particles may be absorbed or degraded by endogenous lytic agents. Risk of recanalization. Best fitted for presurgical embolization purposes rather than stand-alone endovascular curative procedures.

Coils	(i) Detachable coils are most useful for the initial embolization of large fistulae. (ii) Poor penetration potential if compared to particulates or liquid embolic agents-risk of distal dissemination is relatively contained.	(i) Potential for vascular perforation. (ii) Poor penetration potential.
